# MYCT1 Inhibits the Adhesion and Migration of Laryngeal Cancer Cells Potentially Through Repressing Collagen VI

**DOI:** 10.3389/fonc.2020.564733

**Published:** 2021-02-18

**Authors:** Peng-Peng Wang, Si-Yu Ding, Yuan-Yuan Sun, Yun-Hui Li, Wei-Neng Fu

**Affiliations:** ^1^Department of Medical Genetics, China Medical University, Shenyang, China; ^2^Department of Laboratory Medicine, General Hospital of Northern Theater Command, Shenyang, China

**Keywords:** laryngeal cancer, MYCT1, gene interaction network, Collagen Ⅵ, progression

## Abstract

MYCT1, a target of c-Myc, inhibits laryngeal cancer cell migration, but the underlying mechanism remains unclear. In the study, we detected differentially expressed genes (DEGs) from laryngeal cancer cells transfected by MYCT1 using RNA-seq (GSE123275). DEGs from head and neck squamous cell carcinoma (HNSCC) were first screened by comparison of transcription data from the Gene Expression Omnibus (GSE6631) and the Cancer Genome Atlas (TCGA) datasets using weighted gene co-expression network analysis (WGCNA). GO and KEGG pathway analysis explained the functions of the DEGs. The DEGs overlapped between GSE6631and TCGA datasets were then compared with ours to find the key DEGs downstream of MYCT1 related to the adhesion and migration of laryngeal cancer cells. qRT-PCR and Western blot were applied to validate gene expression at mRNA and protein levels, respectively. Finally, the cell adhesion, migration, and wound healing assays were to check cell adhesion and migration abilities, respectively. As results, 39 overlapping genes were enriched in the GSE6631 and TCGA datasets, and most of them revealed adhesion function. Thirteen of 39 genes including COL6 members COL6A1, COL6A2, and COL6A3 were overlapped in GSE6631, TCGA, and GSE123275 datasets. Similar to our RNA-seq results, we confirmed that COL6 is a target of MYCT1 in laryngeal cancer cells. We also found that MYCT1 inhibited the adhesion and migration of laryngeal cancer cells via COL6. These indicate that COL6 is a potential target of MYCT1 and participates the adhesion and migration of laryngeal cancer cells, which provides an important clue for further study on how MYCT1 regulating COL6 in laryngeal cancer progression.

## Introduction

Head and neck squamous cell carcinoma (HNSCC) is the sixth most common malignancy worldwide with approximately 600,000 new cases each year (1). Laryngeal squamous cell carcinoma (LSCC) is a common type of head and neck cancer. Several genes involved in LSCC tumorigenesis and progression might be potential markers for the diagnosis and treatment of laryngeal cancer (2–7).

Metastasis is the leading cause of death in cancer patients, and movement of tumor cells through the extracellular matrix (ECM) is the first stage of invasion. Thus, invasion is characterized by enhanced cell motility, which is caused by alterations in interactions between cells and between cells and the ECM (8). Collagen VI (COL6), a major ECM protein associated with tumors, is a trimeric molecule consisting of three major polypeptide chains, α1 (VI), α2 (VI), and α3 (VI), which are encoded by COL6A1, COL6A2, and COL6A3, respectively (9).

In the previous study, we first cloned MYCT1 in laryngeal cancer, named as c-Myc target from laryngeal cancer (MTLC) (10). We also found that MYCT1 is a down-regulated and suppress cancer cell migration in laryngeal cancer, suggesting that it acts as tumor suppressor (11). Recently, we obtained lots of differentially expressed genes (DEGs) downstream of MYCT1 in laryngeal cancer by transcriptome sequencing (GSE123275). However, the molecular mechanism of MYCT1 in cancer migration invasion has not been delineated. In the present study, we aim to identify DEGS downstream of MYCT1 through analyzing HNSCC gene expression data from the Gene Expression Omnibus (GEO) and The Cancer Genome Atlas (TCGA) together with ours, and explore their function in the adhesion and migration of laryngeal cancer cells.

## Materials and Methods

### Cell Lines and Culture Conditions

The human laryngeal cancer cell line Hep2 was obtained from KeyGen Biotech Company (Nanjing, China). Hep2 cells were cultured in RPMI1640 (GIBCO Thermo Fisher Scientific, MA, USA) supplemented with 10% fetal bovine serum (FBS, Israel), 100 U/ml penicillin, and 100 mg/ml streptomycin (Solarbio, China) in an incubator at 37°C with 5% CO_2_.

### Gene Transfection

The GV230-MYCT1 overexpression plasmids and small interfering RNAs were purchased from Gene Pharma (Shanghai, China). The siRNA sequences are as follows: siMYCT1: 5′-GCUGUGAACGUCGAAGCAATT-3′, siCOL6A2: 5′-GGAGAGUGCUACAAGGUGAGC-3′, siCOL6A3: 5′-GGAGGGAGACAUAGUUCUUGG-3′ negative control RNA: 5′- UUCUCCGAACGUGUCACGUTT-3′. The Hep2 cells were transfected with the relevant GV230-MYCT1 plasmid and siRNAs using jetPRIME^®^ Transfection Reagent (Polyplus-Transfection, Illkirch, France). The cells at 24 or 48 h post transfection were harvested for further use.

### RNA Isolation

Total RNA was extracted from transfected Hep2 cells using RNAiso plus (Takara, Beijing, China). The RNA concentration was measured by reading the absorbance at 260/280 nm.

### RNA-Sequencing and Analysis

RNA sequencing for MYCT1-overexpressing laryngeal cancer cells were carried out by Beijing Genomics Institute (BGI, Shenzhen) with the HiseqTM 4000 PE101 platform. All the gene expression data including those of DEGs were deposited in the GEO database with accession number GSE123275 (https://www.ncbi.nlm.nih.gov/geo/query/acc.cgi?acc=GSE123275). Then DEGs were selected using a multi-hypothesis test correction for *P*-value methods. Summation of log_2_-transformed fold changes higher than 1 and *P*-value lower than 0.05 was used as threshold of the DEGs.

### Data Collection and Pre-Processing

GSE6631 HNSCC gene expression data profiles were obtained from the GEO (https://www.ncbi.nlm.nih.gov/geo) database. GSE6631 is a microarray dataset containing 22 pairs of tumor and adjacent non-tumor HNSCC tissues. The clinical characteristics of the samples are included in the original publication (12). The average transcriptional level of each gene was determined by microarray using multiple probes, and annotations labeled by probes were converted to the same gene ID described in the Ensembl database (http://asia.ensembl.org).

RNA-seq data (level 3) from 43 pairs of tumor and adjacent non-tumor HNSCC tissues and clinical information of the corresponding patients were obtained from the TCGA (http://cancergenome.nih.gov). Each gene was given a sequencing annotation, while was also renamed using their gene ID according to the Ensembl database, and the genes that were not expressed in any of the samples were removed.

### Screening for Differentially Expressed Genes in HNSCC

limma package (http://bioconductor.org/packages/limma) in R-project was applied to identify DEGs by analyzing differences in their transcription levels between tumor and non-tumor tissues from the GSE6631 dataset. Then, the log2 fold change (log2FC) differential expressions and their statistical significance (Benjamini-Hochberg adjusted *P*-values) were calculated using a linear fit model and empirical Bayes method in the limma package of Bionconductor version 3.8. |log2FC| >1 and adjusted *P*-values <0.05 were considered thresholds for screening DEGs in HNSCC. DEGs from GSE6631 were screened out in HNSCC patients and compared with the RNA-seq data (level 3) from the TCGA dataset. Based on the transcription levels of DEGs screened out in each sample, the samples were then clustered to evaluate the sample outliers.

### Identification of Differentially Expressed Genes Related to HNSCC TNM Stages

The WGCNA package in R-project was used to identify HNSCC TNM stage-related DEGs. Based on the transcription levels of DEGs from the GSE6631 dataset, a soft threshold β value was set according to the dynamic threshold to obtain highly related genes, which were clustered to produce signed similarities according to paired Pearson correlation coefficients that were calculated based on gene transcript levels. Scale-free topological overlap matrix (TOM) networks were then generated based on the similarities with 0.25 as the default minimum cluster merge height and 30 as the minimum number of genes to evaluate the correlation among genes in clusters. Based on different correlation values, genes were clustered into different groups marked with different colors that were named as different modules, whereas uncorrelated genes were grouped into the gray module. The related-value and *P*-value of the correlation among genes in each block in the same group were obtained by analyzing their transcription levels when different clinical features were present (13). Based on the transcription levels of DEGs from TCGA datasets, a soft threshold β value, TOM, modules, related-value, and *P*-value of the correlation in each block were obtained by the same analysis mentioned above to further confirm the results from the GSE6631 dataset.

A cross-comparison of DEGs in each module was established using the GSE6631 dataset and the TCGA dataset except for the genes in the gray module. The DEGs were then applied to gene ontology (GO) and Kyoto Encyclopedia of Genes and Genomes (KEGG) pathway analyses according to the DAVID (https://david.ncifcrf.gov) (14, 15) and the ClueGO application in Cytoscape version 3.42 (16). In the ClueGO application of Cytoscape, GO based on GO-BiologicalProcess-EBI-QuickGO-GOA 12.01.2018 and KEGG based on KEGG 12.01.2018.

### qRT-PCR Detection

RNA was reverse-transcribed to cDNA using a HiScript® II Q RT SuperMix for qPCR (Vazyme Biotech, Nanjing, China). Real-time PCR was performed using TB Green^TM^ Premix Ex Taq^TM^ II (Takara, Beijing, China) under the following conditions: (a) 95°C for 30 s, (b) 40 cycles of 95°C for 5 s and 60°C for 34 s, and (c) 95°C for 15 s, 60°C for 1 min, and then 95°C for 15 s. The relative transcript expression of each gene normalized to glyceraldehyde-3-phosphate dehydrogenase (GAPDH) was calculated using the 2^−ΔΔCt^ method. Primer sequences of the amplified genes are listed in **Supplementary File 3**.

### Western Blot Analysis

Protein was isolated from cells and subjected to sonication in ice-cold lysis buffer. Denatured protein was separated on sodium dodecyl sulfate-polyacrylamide gel and transferred to a polyvinylidene fluoride (PVDF) membrane (Millipore, MA, USA). After blocking for 2 h at room temperature, the PVDF membrane was incubated with anti-Collagen Type VI [Proteintech, CHI, USA (17023-1-AP)], anti-MYCT1 [Abcam, Cambridge, UK (ab139945)], and anti-β-actin [Cloud-Clone Corp. USA (CAB340Mi22)] primary antibodies, followed by incubation with appropriate peroxidase-coupled secondary antibodies. Proteins on the PVDF membrane were stained with ECL (WanleiBio, Shenyang, China) and visualized using ChemiDoc^TM^ Touch Imaging System (Bio-Rad, CA, USA). The relative protein expression of each gene normalized to β-actin was calculated based on integrated band density by Image Lab software (Bio-Rad, CA, USA).

### Cell Adhesion Assay

The cell adhesion assay was performed as described below. Briefly, each well of a flat bottom 96-well plate was coated with 50 µl of Matrigel (0.04 µg/ml; Corning Life Sciences, NY, USA) in RPMI1640 after the plate was incubated at 37°C for 1 h. Each well was blocked with 0.1% BSA at 37°C for 1 h followed by three washes in RPMI1640 medium. The cells were harvested by trypsin/EDTA digestion, washed twice, resuspended in RPMI1640 medium, and labeled by 2 µM Calcein AM (KeyGen Biotech Company, Nanjing, China) at 37°C for 30 min. Labeled cells (2 × 10^4^ cells/well) were transferred to each well and incubated at 37°C for 2 h. Cells in each well were washed three times with RPMI1640 medium to remove nonadherent cells. The fluorescence of adherent cells in each well was measured with a Spark multimode microplate reader (TECAN, Austria) at 490 nm. The percentage of the adherent cells in each well was calculated and normalized to the number of initial input cells.

### Cell Migration Assay

Cell migration was assayed using a 24-well Transwell chamber without Matrigel. Briefly, cells were resuspended in serum-free medium and then seeded in the top chamber. The lower chamber was filled with culture medium containing 10% FBS. After incubation at 37°C for 24 h, cells in the lower chamber were fixed in 4% paraformaldehyde and stained with hematoxylin and eosin. The migrated cells were visualized and counted in randomly selected fields under a microscope (Leica DM4000 B LED) and analyzed by LAS X software (Leica Microsystems, Wetzlar, GER).

### Wound Healing Assay

Cells were cultured in six-well plates in serum-free medium and were scraped with a 200 µl sterile pipette tip at 0 h. Wound healing images were then captured with a Leica DMI3000 B microscope at 0, 12, and 24 h, and then analyzed using LAS AF software (Leica Microsystems, Wetzlar, GER).

### Statistical Analysis

Each experiment was performed at least three times. Data were analyzed by two-tailed Student’s *t*-test or one-way analysis of variance using GraphPad Prism 6.01 (GraphPad Software Inc., CA, USA). Data are expressed as the mean ± standard deviation. *P* <0.05 was considered statistically significant.

## Results

Our RNA-seq results revealed 4,227 DEGs downstream of MYCT1 (GSE123275). In order to find the key DEGs downstream of MYCT1 associated with laryngeal cancer migration and metastasis, we first identified the DEGs in HNSCC by data mining.

### Identification of Gene Modules Related to TNM Stage

**Figure 1:**
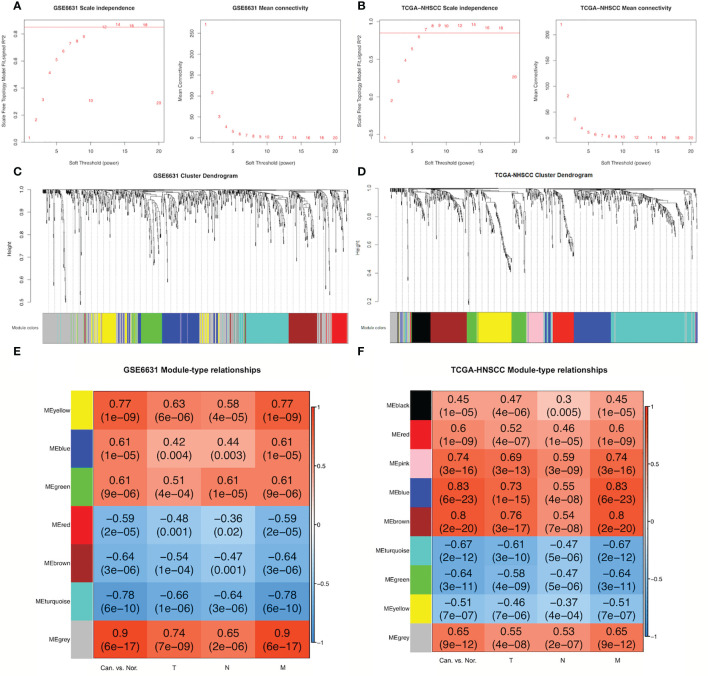
Construction of GSE6631- and TCGA-HNSCC-specific modules using WGCNA. **(A)** Network topology for different soft-thresholding powers of GSE6631. Left: the scale-free topology fit index (y-axis) in the GSE6631 dataset; right: the mean connectivity (y-axis) as a function of the soft-thresholding power (x-axis) in the GSE6631 dataset. **(B)** Network topology for different soft-thresholding powers of TCGA-HNSCC. Left: the scale-free topology fit index (y-axis) in the TCGA-HNSCC dataset; right: the mean connectivity (y-axis) as a function of the soft-thresholding power (x-axis) in the TCGA-HNSCC dataset. **(C)** Branches of the clustering dendrogram from different modules based on differentially expressed genes (DEGs) in GSE6631. Based on the unsupervised hierarchical clustering, GSE6631 DEGs were clustered into seven modules (yellow, blue, green, red, brown, turquoise, and gray). **(D)** Branches of the clustering dendrogram from different modules based on DEGs in TCGA-HNSCC. Based on the unsupervised hierarchical clustering, TCGA-HNSCC DEGs were clustered into nine modules (black, red, pink, blue, brown, turquoise, green, yellow, and gray). **(E)** Module-clinical feature relationships in the GSE6631 modules. In the heatmap, red represents a positive correlation, whereas blue represents a negative correlation. The numbers in each block are the related-value and *P*-value of the correlation. **(F)** Module-clinical feature relationships in TCGA-HNSCC modules. In the heatmap, red represents a positive correlation, and blue represents a negative correlation. The numbers in each block are the related-value and *P*-value of the correlation.

Based on the thresholds |log2FC| >1 and adjusted *P-*value <0.05, 903 DEGs were found in microarray data from the GSE6631-HNSCC dataset whereas 839 in the TCGA-HNSCC RNA-seq dataset (**Supplementary File 1**). In order to set up GSE6631- and TCGA-modules related to TNM stage, scale independence from GSE6631 and TCGA datasets was set at 0.85, soft thresholds for the GSE6631 and TCGA datasets were 12 and 7, respectively, and both datasets obtained higher mean connectivity (**Figures 1A**, **B**). Cluster dendrogram results indicated that 903 DEGs from the GSE6631 dataset and 839 DEGs from the TCGA dataset were grouped into 7 and 9 modules, respectively (**Figure 1C**).

The relationships between DEGs from different modules and clinical features including HNSCC vs. normal tissues and TNM stages were analyzed. As a result, three modules (yellow, blue, and green) from the GSE6631 dataset showed significantly positive correlation with clinical features (r > 0.3, *P* < 0.05), whereas three (red, brown, and turquoise) significant negative correlation (r > –0.3, *P* < 0.05). Meanwhile, eight modules (black, red, pink, blue, brown, turquoise, green, and yellow) from the TCGA dataset revealed significant correlation with clinical features, five of which were positive, whereas three were negative (**Figures 1C–F**).

### Identification of Overlapping Genes Related to the Clinical Features of HNSCC

**Figure 2:**
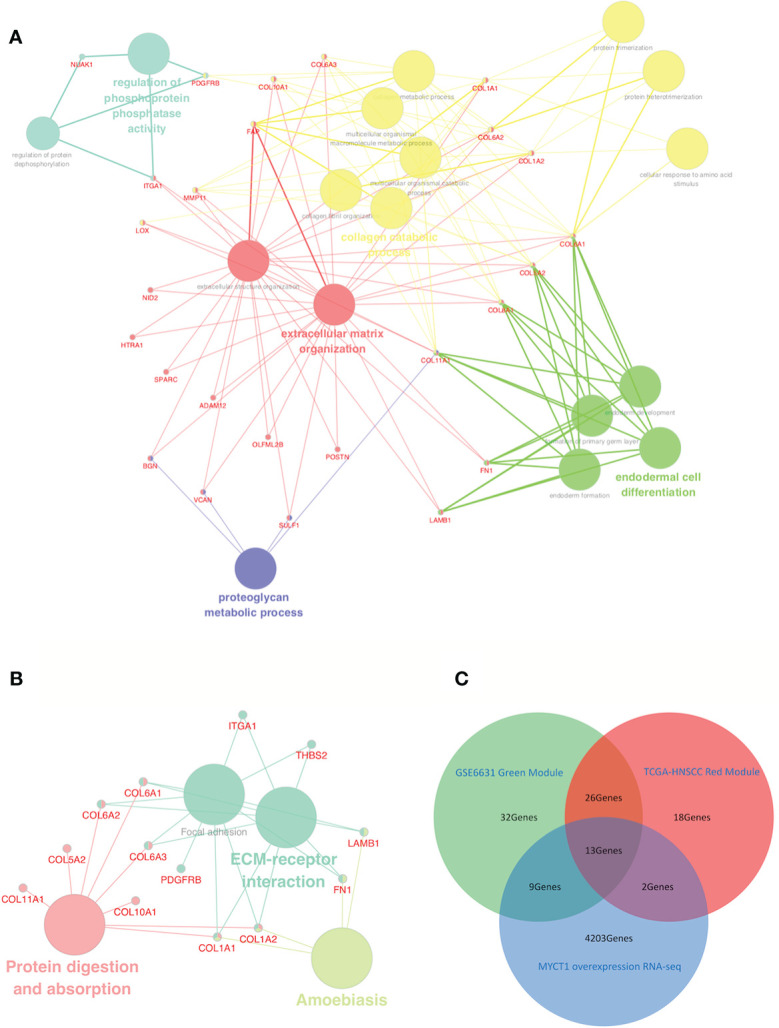
Identification of a functional network and laryngeal cancer-associated DEGs from the red-green overlapping DEGs. **(A)** GO term interactions among overlapping DEGs. **(B)** KEGG pathway interactions among overlapping DEGs. **(C)** Venn diagram of laryngeal cancer associated DEGs downstream of MYCT1 from the GSE123275, GSE6631, and TCGA datasets. The blue circle represents the unique DEGs in the GSE6631 green module. The yellow circle represents the DEGs in the TCGA-HNSCC red modules. Large nodes represent GO terms **(A)** or KEGG pathways **(B)**, whereas small nodes represent the DEGs. The colors of the nodes represent different GO terms or KEGG pathways in each network diagram. The red circle represents the DEGs downstream of MYCT1 in Hep2 cells. In the middle area, 13 genes were distinguished out of three datasets.

Based on gene modules except for the gray ones, a conjoint analysis of all genes between two groups of modules was performed. As a result, all genes in the modules displayed either positive or negative correlations. All positive correlations but not negative ones between modules from each group showed highly repetitive rates (**Supplementary File 1**), suggesting that negative correlations are not considered in this study. Based on genes in the modules with positive correlations, the most highly repetitive rates were 49% in the green module from the GSE6631 dataset and 66% in the red module from the TCGA dataset. Thirty-nine genes having the similar expression pattern between the two modules was observed (**Supplementary File 1**, **Figure 2**), which were the focus of the following study.

### Enrichment of Signaling Pathways and Functions Points to Adhesion

GO analysis results revealed that 26 of 39 overlapping genes which were involved in 17 functions (**Supplementary File 1**, **Figure 2A**). In the KEGG pathway analysis, 13 of 39 genes were enriched in ECM–receptor interaction, amebiasis, focal adhesion, protein digestion and absorption pathways (**Figure 2B**). By comparison of the GO terms and the KEGG pathways, most genes including a set of Collagen VI family members were enriched in adhesion function for both GO terms and KEGG pathways, which suggest that adhesion should be the focus of the following study (**Figures 2A, B**).

### Collagen Ⅵ Family Members are Important Mediators in the MYCT1-Associated Migration of Laryngeal Cancer Cells

We then compared overlapping genes among the GSE123275, GSE6631, and TCGA datasets. As a result, 13 genes were overlapped, 5 of which belong to collagen family members, including COL6A1, COL6A2, and COL6A3 (**Figure 2C**, **Supplementary File 1**), which implies that the 13 genes, especially Collagen VI family members, play importantly potential roles in laryngeal cancer cell migration.

### COL6 was Confirmed as a Target Downstream of MYCT1 in Laryngeal Cancer Cells

**Figure 3:**
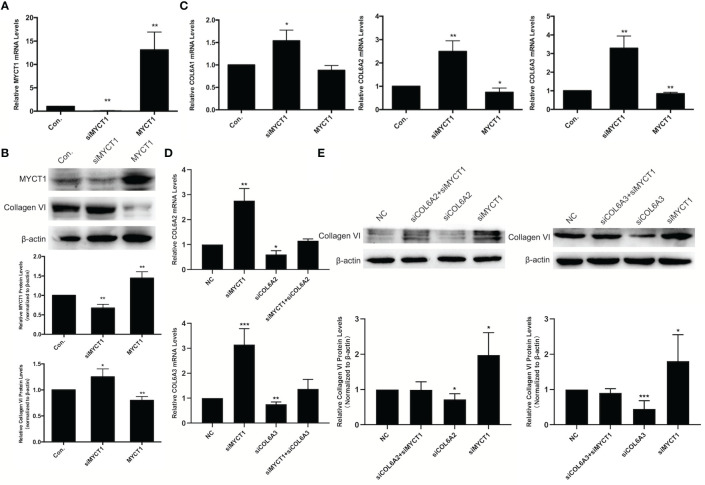
MYCT1 downregulates COL6A1, COL6A2, and COL6A3 gene expression. **(A)** MYCT1 mRNA level in Hep2 cells transfected with MYCT1 and siMYCT1. **(B)** Effect of MYCT1 on the levels of COL6A1, COL6A2, and COL6A3 mRNA. The levels of COL6A1, COL6A2, and COL6A3 mRNA were detected by qRT-PCR in Hep2 cells transfected with control, siMYCT1 RNA, or MYCT1. **(C)** Effect of MYCT1 on the Collagen VI protein level. MYCT1 and Collagen VI protein level in Hep2 cells transfected with MYCT1 and siMYCT1. The Collagen VI protein level was examined by Western blot in Hep2 cells transfected with control, siMYCT1 RNA, or MYCT1. **(D)** Effects of MYCT1 knockdown on COL6A2 and COL6A3 mRNA expression levels in Hep2 cells by qRT-PCR **(E)** Effects of MYCT1 knockdown on COL6A2 and COL6A3 protein levels in Hep2 cells by western bolt. Symbols *, ** and *** indicate P < 0.05, P < 0.01, and P < 0.001, respectively.

In our RNA-seq results, COL6A1, COL6A2, and COL6A3 were downregulated at mRNA levels in MYCT1 overexpressing laryngeal cancer cells (GSE123275). As shown in **Figures 3A, B**, MYCT1 was significantly decreased and increased both at mRNA and protein levels in laryngeal cancer cells transfected with siMYCT1 and MYCT1 compared with controls (*P* < 0.01), respectively, suggesting that transfection is successive. At the protein level, Collagen VI was significantly upregulated and downregulated in siMYCT1- and MYCT1-transfected laryngeal cancer cells compared with controls (*P* < 0.01, **Figure 3B**), respectively. At the mRNA level, Knockdown of MYCT1 significantly increased the COL6A1, COL6A2, and COL6A3 expression but MYCT1 decreased the COL6A2, and COL6A3 expression in laryngeal cancer cells (*P* < 0.01, **Figure 3C**), respectively. We also found that both COL6A2 and COL6A3 knockdown significantly recued the effects of MYCT1 silence on their expressions in laryngeal cancer cells (*P* < 0.01, **Figures 3D, E**), respectively. These results confirmed that COL6 is a target of MYCT1 in laryngeal cancer cells.

### MYCT1 Inhibits the Adhesion and Migration of Laryngeal Cancer Cells *via* COL6

**Figure 4:**
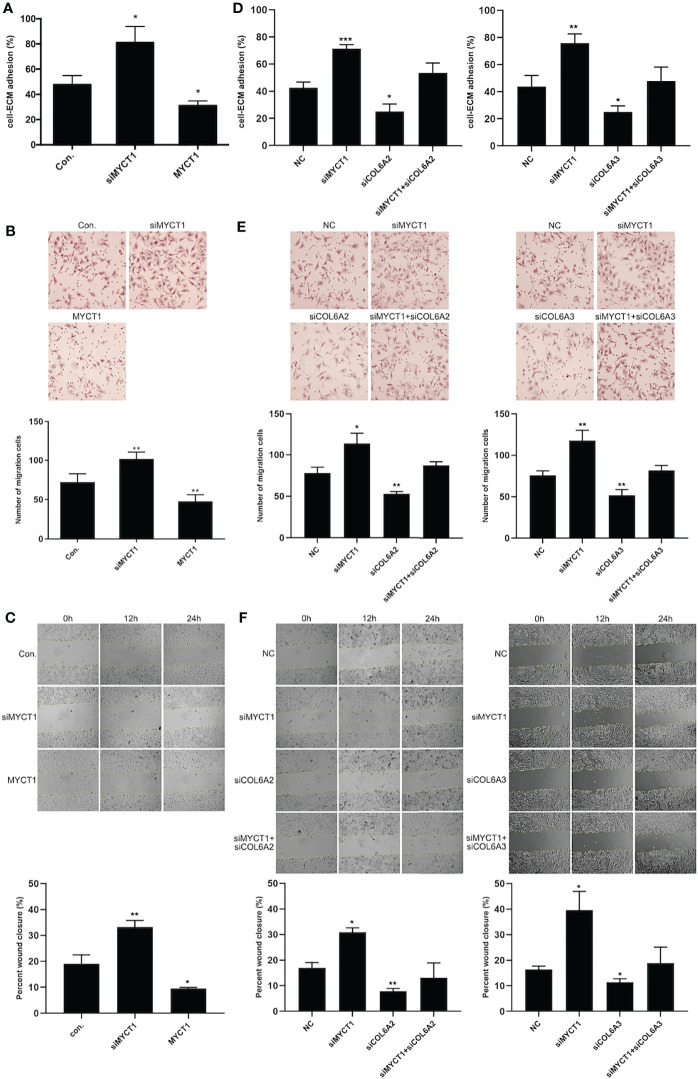
MYCT1 inhibits laryngeal cancer cell migration and adhesion. **(A)** Effect of MYCT1 on laryngeal cancer cell adhesion by cell adhesion assay. **(B)** Effect of MYCT1 on laryngeal cancer cell migration according to a Transwell experiment. **(C)** Effect of MYCT1 on laryngeal cancer cell migration according to a wound healing assay. **(D)** Effect of MYCT1 knockdown on COL6A2 and COL6A3 in laryngeal cancer cell adhesion by cell adhesion assay. **(E)** Effect of MYCT1 knockdown on COL6A2 and COL6A3 in laryngeal cancer cell migration according to a Transwell experiment. **(F)** Effect of MYCT1 knockdown on COL6A2 and COL6A3 in laryngeal cancer cell migration according to a wound healing assay. Symbols *, ** and *** indicate *P* < 0.05, *P* < 0.01, and *P* < 0.001, respectively.

MYCT1 knockdown and overexpression significantly increased and decreased the adhesion, migration and wound healing abilities of the laryngeal cancer cells compared with controls (*P* < 0.01, **Figures 4A–C**, **Supplementary File 2A**), respectively. As shown in **Figures 4D–F** and **Supplementary File 2A**, COL6A2 and COL6A3 knockdown significantly recued the effects of MYCT1 silence on the adhesion, migration and wound healing abilities of the laryngeal cancer cells (*P* < 0.01), respectively. These results indicated that MYCT1 suppresses the adhesion and migration of laryngeal cancer cells via COL6.

## Discussion

Some cellular processes, such as ECM degradation and epithelial-to-mesenchymal transition, are essential for cancer metastasis (17). It is generally accepted that cancer invasion and metastasis involve tumor cell adhesion changes, ECM degradation, and cancer cell migration in a proteolytically modified ECM (18). ECM not only acts as a scaffold to which cells are anchored but also plays a significant role in cell function and behavior (19).

COL6 is a major ECM-associated protein in the tumor microenvironment (20, 21). COL6 also forms a discrete network of beaded microfilaments, which interact with other ECM molecules and contribute to structural support for cells (22). COL6 is highly expressed in a series of cancer and promotes cancer cell growth and metastasis to directly or indirectly accelerate cancer progression by educating other microenvironmental components (21, 23).

WGCNA is an innovative statistical tool used to cluster sets of genes with similar expression patterns and to analyze the correlation between genes and clinical features. WGCNA has been widely used in the analysis of cancer, such as lung (24), breast (25), colorectal (26), liver (27), thyroid (28), and HNSCC (29–31). However, WGCNA-related studies on laryngeal cancer metastasis have seldom been reported.

In this study, we found 903 and 839 DEGs from the GSE6631 and TCGA datasets, respectively, which were grouped into 7 and 9 modules, respectively. Thirty-nine DEGs were present in both datasets, 13 of which, including COL6A1, COL6A2, and COL6A3, were enriched in ECM-receptor interaction, amebiasis, focal adhesion, protein digestion, and absorption pathways. In MYCT1-overexpressing laryngeal cancer cells, another 13 DEGs (GSE123275) were overlapped in the GSE6631 and TCGA datasets, where COL6A1, COL6A2, and COL6A3 were also present. This implies that COL6A1, COL6A2, and COL6A3 are crucial in laryngeal cancer.

In our previous study, RNA-seq detection showed that the significantly low expression of COL6A1, COL6A2, and COL6A3 in MYCT1-overexpressing laryngeal cancer cells that was confirmed in the study, which suggests that the three COL6 members are potential targets downstream of MYCT1 in laryngeal cancer. However, the mechanism is still unclear.

In the study, we also found that MYCT1 inhibits laryngeal cancer cell adhesion and migration and the effects are rescued by Collagen VI. These findings indicate that MYCT1 participates in the regulation of laryngeal cancer cell adhesion and migration at least via downregulating the COL6 gene expression. Previously, we discovered that MYCT1 suppresses laryngeal cancer cell migration through the CREB/MYCT1/NAT10 axis (32). But MYCT1-mediated laryngeal cancer cell adhesion has not been reported. Therefore, we plan to focus on the following two issues in future studies: 1) regulatory mechanism of MYCT1 on COL6; 2) molecular mechanism of COL6 in laryngeal cancer cell adhesion and migration.

## Conclusions

We first found that a set of DEGs such as COL6A1, COL6A2, and COL6A3 are probably involved in the progression laryngeal cancer and MYCT1 inhibits the adhesion and migration of laryngeal cancer cells potentially through repression of COL6, suggesting that COL6 is a target downstream of MYCT1 and contributes to the progression of laryngeal cancer.

## Data Availability Statement

The datasets presented in this study can be found in online repositories. The names of the repository/repositories and accession number(s) can be found below: https://www.ncbi.nlm.nih.gov/geo/, GSE6631 and GSE123275; http://cancergenome.nih.gov, RNA-seq data (level 3).

## Author Contributions

Conceived and designed the experiments, P-PW, W-NF. Performed the experiments analyzed the data, P-PW, S-YD. Contributed reagents, Y-YS. Materials and analysis tools, Y-HL. Contributed on the writing of the manuscript P-PW, W-NF. All authors contributed to the article and approved the submitted version.

## Funding

This work was supported by the National Natural Science Foundation of China (81372876), Liaoning Science and Technology Project (2017020201-301, LQNK201726).

## Conflict of Interest

The authors declare that the research was conducted in the absence of any commercial or financial relationships that could be construed as a potential conflict of interest.

## Supplementary Material

Supplementary File 1 List of differentially expressed genes in different datasets.

Supplementary File 2 High resolution of **Figures 4C** and **4D**.

Supplementary File 3 Primer sequences used in the study.
