# Supplementary material for: MYCT1 Inhibits the Adhesion and Migration of Laryngeal Cancer Cells Potentially Through Repressing Collagen VI
Source: Front Oncol. 2021 Feb 18;10:564733. doi: 10.3389/fonc.2020.564733 (PMC7931689; doi:10.3389/fonc.2020.564733)
Supplement: Supplementary File 2 — High resolution of Figures 4C and 4D. [file DataSheet_2.pdf]

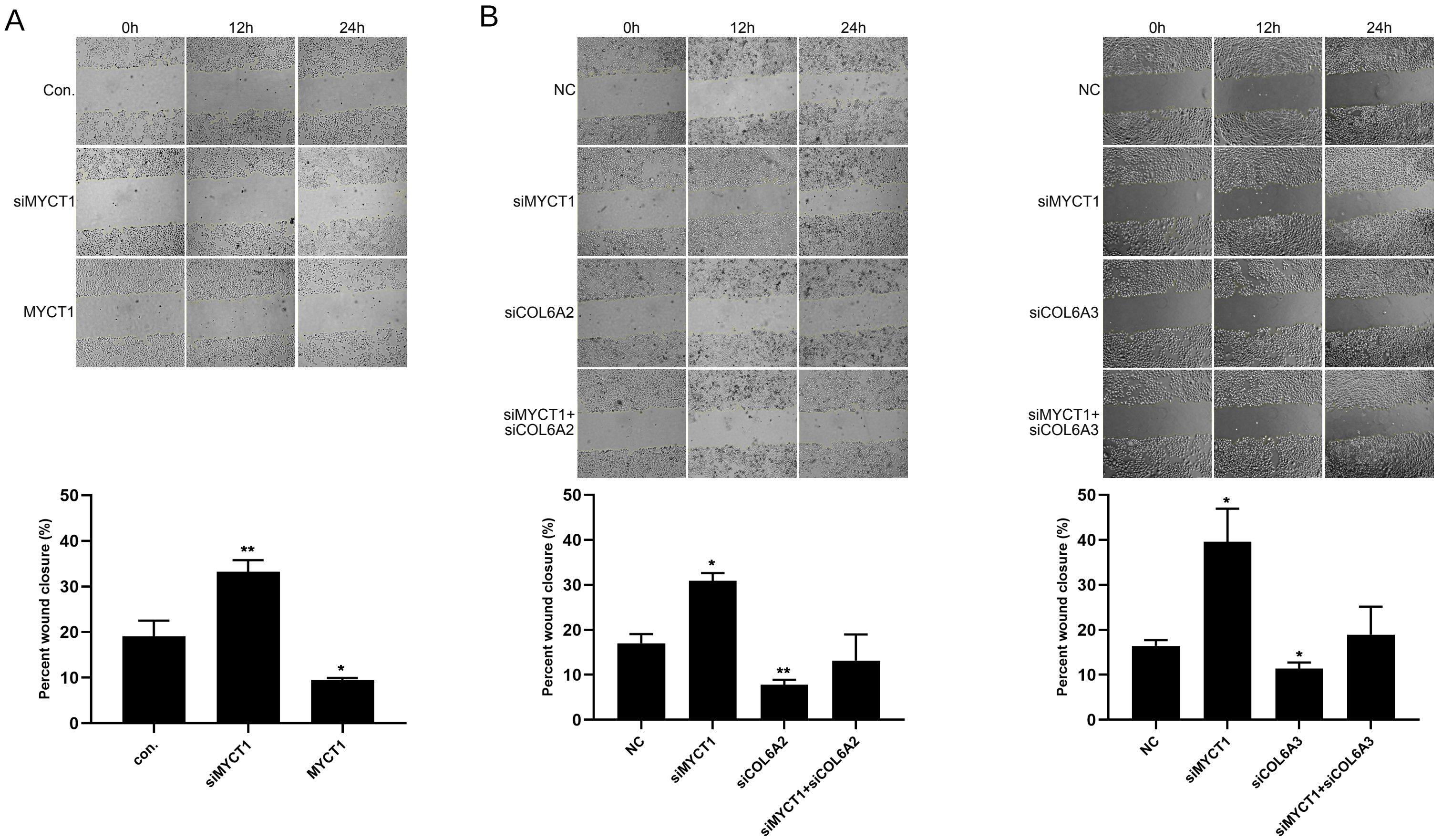

Supplementary Figure 1：(A) Effect of MYCT1 on laryngeal cancer cell migration according to a wound healing assay. (B) Effect of MYCT1 knockdown on COL6A2 and COL6A3 in laryngeal cancer cell migration according to a wound healing assay. Symbols *, ** and *** indicate $P < 0.05$, $P < 0.01$ and $P < 0.001$ respectively.
