# Supplementary material for: MYCT1 Inhibits the Adhesion and Migration of Laryngeal Cancer Cells Potentially Through Repressing Collagen VI
Source: Front Oncol. 2021 Feb 18;10:564733. doi: 10.3389/fonc.2020.564733 (PMC7931689; doi:10.3389/fonc.2020.564733)
Supplement: Supplementary File 3 — Primer sequences used in the study. [file DataSheet_3.docx]

Supplementary 3: Primer sequences used in the study

| **Name** | **Sequence** |
| --- | --- |
| MYCT1-Forward primer | 5′-GCCAGAAAACTTTTGGGAGGA-3′ |
| MYCT1-Reverse primer | 5′-ATCCAGTTCTGTTGAGGCCG-3′ |
| GAPDH-Forward primer | 5′-TGGCACCCAGCACAATGAA-3′ |
| GAPDH-Reverse primer | 5′-CTAAGTCATAGTCCGCCTAGAAGCA-3′ |
| COL6A1-Forward primer | 5′-TCAAGAGCCTGCAGTGGATG-3′ |
| COL6A1-Reverse primer | 5′-TGGACACTTCTTGTCTATGCAG-3′ |
| COL6A2-Forward primer | 5′-TATAGGATCCTTCGTCATCAACGTGGTCAAC-3′ |
| COL6A2-Reverse primer | 5′-TATAGAATTCCTAGCAGATCCAGCGGATGAA-3′ |
| COL6A3-Forward primer | 5′-TCTCTTAAAATCAGTGCACAACG-3′ |
| COL6A3-Reverse primer | 5′-AACTCTTTCAACAGAGGGAAGC-3′ |
